# Elasto-Inertial Focusing Mechanisms of Particles in Shear-Thinning Viscoelastic Fluid in Rectangular Microchannels

**DOI:** 10.3390/mi13122131

**Published:** 2022-12-01

**Authors:** Mohammad Moein Naderi, Ludovica Barilla, Jian Zhou, Ian Papautsky, Zhangli Peng

**Affiliations:** Department of Biomedical Engineering, University of Illinois Chicago, 851 S. Morgan Street, 218 SEO, Chicago, IL 60607, USA

**Keywords:** viscoelastic microfluidics, elasto-inertial focusing, particle separation, shear-thinning, *Giesekus* model

## Abstract

Growth of the microfluidics field has triggered numerous advances in focusing and separating microparticles, with such systems rapidly finding applications in biomedical, chemical, and environmental fields. The use of shear-thinning viscoelastic fluids in microfluidic channels is leading to evolution of *elasto-inertial* focusing. Herein, we showed that the interplay between the elastic and shear-gradient lift forces, as well as the secondary flow transversal drag force that is caused by the non-zero second normal stress difference, lead to different particle focusing patterns in the *elasto-inertial* regime. Experiments and 3D simulations were performed to study the effects of flowrate, particle size, and the shear-thinning extent of the fluid on the focusing patterns. The *Giesekus* constitutive equation was used in the simulations to capture the shear-thinning and viscoelastic behaviors of the solution used in the experiments. At low flowrate, with *Weissenberg* number *Wi* **~** O(1), both the elastic force and secondary flow effects push particles towards the channel center. However, at a high flowrate, *Wi* **~** O(10), the elastic force direction is reversed in the central regions. This remarkable behavior of the elastic force, combined with the enhanced shear-gradient lift at the high flowrate, pushes particles away from the channel center. Additionally, a precise prediction of the focusing position can only be made when the shear-thinning extent of the fluid is correctly estimated in the modeling. The shear-thinning also gives rise to the unique behavior of the inertial forces near the channel walls which is linked with the ‘warped’ velocity profile in such fluids.

## 1. Introduction

Microfluidics has a wide range of applications in a variety of research fields and industries, including biomedical, chemical, and environmental engineering [1,2,3]. While nanofluidic structures mainly focus on the enhancement of thermal conductivity by altering thermophysical properties of the fluid [4,5], Microelectromechanical Systems (MEMS) possess versatile capabilities, making them suitable for implementation as biosensors [6], resonators in digital circuits [7], and controllable drug delivery systems [8]. More importantly, microfluidics affords the ability to precisely manipulate fluids, particles, and cells owing to the laminarity of the flow inside microchannels [9]. Therefore, microfluidic chips have been used extensively over the past two decades to mix, sort, capture, focus, and separate particles and cells [10,11,12,13,14]. In general, these devices can be categorized as active or passive [15]. While active microfluidic methods take advantage of external force fields such as acoustic [16,17], magnetic [18,19,20], and electric fields [21,22], passive techniques rely solely on either channel geometry [23,24], particle size [15,25,26], shape [27] and deformability [28]. Deterministic lateral displacement (DLD) [29,30], microfiltration [23,31,32], and inertial focusing [33,34,35] are among the most commonly used passive separation methods. We recently reviewed this topic and the latest advances in label-free microfluidic sorting devices [36]. Although these methods can achieve high quality focusing and separation, limitations in throughput of active separation devices [37] and clogging issues in microfiltration processes [23], brought increased attention to inertial focusing as a reliable, high-throughput, and robust separation technique.

Inertial focusing of particles was originally reported by Segrè and Silberberg in 1961 [38], who observed that a uniformly distributed particle population introduced to the inlet of a cylindrical pipe formed a thin annular ring at the outlet. Owing to significant experimental and computational advances in the past two decades, rigorous mechanisms have been proposed to explain the underlying physics behind inertial motion of particles in microchannels [26,39,40]. These studies have shown that particle streamwise migration in *Newtonian* fluid is mainly due to the combined effects of the wall-induced (F_W_) and shear-gradient (F_S_) lift forces. While F_W_ pushes the particles away from the channel walls, F_S_ generates a wall-directed force due to the parabolic nature of the velocity profile, and the final equilibrium position of particles is determined by the interplay of these two forces. These forces are present in flows with low but finite Reynolds number [41], which is a dimensionless parameter that is the ratio of inertial force to viscous force in the flow and is defined as *Re* = ρVD_H_/μ where ρ is the fluid density, V is the characteristic velocity, D_H_ is the hydraulic diameter and μ is the fluid viscosity. In the case of a square channel, particles occupy four focusing positions along the channel walls at the center of each face, while in a rectangular channel, two focused streams are formed near centerline of the wider channel walls. Thus, single-stream focusing for cytometry or size-based separation is not feasible in simple, straight microchannels using *Newtonian* fluids and instead requires more complex channel geometries [33,42,43].

**Figure 1:**
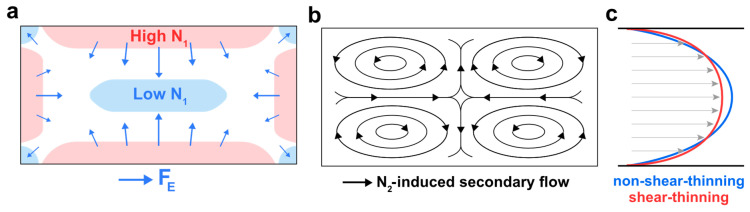
Schematics of the viscoelastic phenomenon in a rectangular channel flow of *Giesekus* fluid. (**a**) First normal stress difference (N_1_) distribution in the cross-section; Elastic force drives particles towards the lowest N_1_ regions. (**b**) Elasticity-induced secondary flow due to the presence of the non-zero second normal stress difference (N_2_). (**c**) Comparison between the velocity profile in non-shear-thinning fluids (*Newtonian*, *Oldroyd-B*, etc.) and the warped velocity profile in shear-thinning fluids (*Giesekus*, etc.).

Viscoelastic fluids have been introduced in microfluidics as a substitute for *Newtonian* fluids to enable focusing of particles in single streams and allow improved size-based separations in straight channels [44]. Significant efforts have been made in recent years to understand the effects of viscoelasticity on particle migration. Particles experience additional elastic force (F_E_) attributed to the unique properties of viscoelastic flows, namely the first and second normal stress differences (N_1_ and N_2_). Studies show that F_E_ drives particles towards regions with the lowest N_1_ (Figure 1a), while N_2_ gives rise to the formation of secondary flows in the cross-section of the channel (Figure 1b), which can in turn affect particle lateral migration [45] by imposing a transversal drag force, F_SFD_. Additionally, shear-thinning properties will lead to decrease in the local fluid viscosity near the channel walls and its increase near the channel center. This gives rise to the unique ‘warped’ velocity profile in such flows, yielding velocity gradient that is more flattened at the center and sharper near the channel walls (Figure 1c)**.** Viscoelasticity of the fluid can thus be characterized by the Weissenberg (*Wi*) dimensionless number, defined as the ratio of elastic force to viscous force, *Wi* = $\lambda \dot{\gamma }$, where λ is the relaxation time of the fluid, and $\dot{\gamma }$ is the shear rate.

*Elasto-inertial* focusing occurs when particles are under combined effects of the inertial and elastic forces. For example, Yang et al. [46] found that single stream focusing of particles is possible in flows where both *Re* and *Wi* numbers are finite and the elasticity number, defined as *El* = *Wi/Re*, is **~** *O*(1–10). A summary of the related published works is given in Table 1. These studies provide invaluable insight into the physics behind viscoelastic focusing mechanisms in a wide range of channel cross-sectional shapes and flow regimes. Nevertheless, a more detailed study on the elasto-inertial focusing mechanisms of shear-thinning fluids in rectangular microchannels is indeed needed at higher Wi numbers, since most numerical investigations in Table 1 are limited to *Wi* < 3 due to convergence issues arising at higher *Wi* numbers [47]. In addition, in most of these works, the contributions of shear-thinning and elastic forces were studied separately and the combined effects were not systematically explored. Moreover, most of these studies are either purely experimental or computational studies, and systematic comparison between experiments and models for elasto-inertial focusing is lacking.

In this work, we use both experimental and computational methods to understand how focusing position of particles along the two midlines of a rectangular channel alters as a function of fluid viscoelasticity, flowrate, and particle size. Fluid properties are chosen such that the elasticity number is fixed at *El* = 18 to ensure the *elasto-inertial* regime. Using the *Giesekus* constitutive equation, we explore how shear-thinning and N_2_-induced secondary flow modify the inertial and elastic forces and give rise to unique focusing patterns across channel centerlines. We then propose a general focusing mechanism based on our experimental and numerical observations. Ultimately, our results show that precise prediction of focusing positions, which has crucial impact on the efficiency of particle separation, is possible, but only when realistic constitutive equations and parameters are utilized in the simulations. Lastly, we show how the ‘warped’ velocity profile in shear-thinning fluids impacts the inertial force behavior near the channel walls.

## 2. Methods

### 2.1. Experimental Methods

#### 2.1.1. Device Fabrication

A 50 µm × 25 µm (width × height) microchannel was fabricated in polydimethylsiloxane (PDMS) using a dry film master. The channel was 40 mm in length. The process for making the dry film master is detailed in our recent work [56]. Briefly, dry films (ADEX 50, DJ MicroLaminates Inc., Sudbury, MA, USA) were used to pattern the microchannel on a 3″ silicon wafer by conventional photolithography. PDMS monomer and curing agent (Sylgard 184, Dow Corning^®^, Midland, MI, USA) were mixed with a ratio of 10:1. The mixture was cast on the dry film wafer after degassing for 30 min. The PDMS slab containing the replicated microchannel was peeled after 2 h curing on an 80 °C hotplate. A biopsy punch with an outer diameter of 1.5 mm (Ted Pella Inc., Redding, CA, USA) was used to manually punch the inlet and outlet holes in the PDMS channel, which was subsequently bonded to 1″ × 3″ glass slides (Fisher Scientific, Hampton, NH, USA) after O_2_ surface plasma treatment (PE-50, Plasma Etch Inc., Carson City, NV, USA) for 20 s.

#### 2.1.2. Particle Sample Preparation

Polyethylene oxide (PEO) powder with molecular weight of 2 × 10^6^ g/mol (Sigma-Aldrich, Burlington, MA, USA) was dissolved into distilled water to create a viscoelastic carrier fluid. The concentration of PEO was 0.1% (*wt*/*wt*) for the 1000 ppm solution. Fluorescent polystyrene particles with 4.16 µm and 7.32 µm diameter were suspended into the PEO solutions at a volume fraction of 0.01% to avoid potential particle-particle interactions. A drop (1% *v*/*v*) of Tween 80 (Sigma-Aldrich, Burlington, MA, USA) was added to the suspensions to reduce particle aggregation. According to Li et al. [48], the zero-shear viscosity ($\mu_{0}$) for the 1000 ppm PEO solution is 2.3 mPa·s. The corresponding effective relaxation time and the density of the PEO solution are 6.8 ms, and 1000 kg/m^3^, respectively.

#### 2.1.3. Flow Experiments

Sample solution was loaded in a 5 mL syringe (Norm-Ject^®^, Air-Tite Co Inc, Virginia Beach, VA, USA), which was connected to 1/16″ Tygon^®^ tubing (Cole-Palmar, Vernon Hills, IL, USA) using proper fittings (IDEX Health & Science LLC, Northbrook, IL, USA). The other end of the tubing was secured to the device inlet. A syringe pump (Legato 200, KD Scientific Inc, Holliston, MA, USA) was used to sustain various stable flow rates (1 & 5 µL/min).

#### 2.1.4. Data Acquisition and Analysis

The microchannel was placed on the stage of an inverted epi-fluorescent microscope (IX83, Olympus America, Center Valley, PA, USA). Fluorescent images of particle flow inside the microchannel were acquired using a high-resolution sCMOS camera (Andor Zyla 5.5, Oxford Instruments, Santa Barbara, CA, USA) mounted on the microscope along with the CellSense imaging software (Olympus America, Center Valley, PA USA). At least 150 fluorescent images were acquired at the channel outlet for every flow rate. The fluorescent images were stacked to generate composite micrographs of particle streaks. Bright-field images were obtained using a high-speed camera (Photron Mini AX200, Photron USA. Inc., San Diego, CA, USA). All images were analyzed using ImageJ^®^ (NIH, Bethesda, MD, USA).

### 2.2. Numerical Methods

#### 2.2.1. Direct Numerical Simulation (DNS)

**Figure 2:**
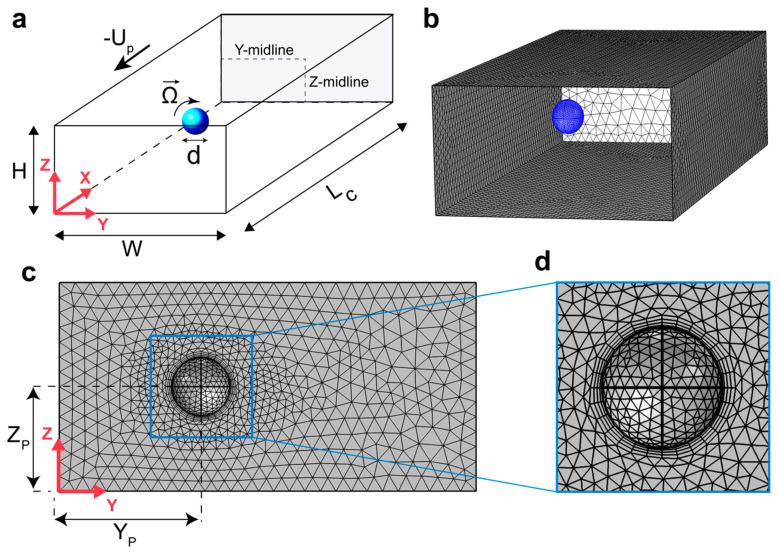
Model schematics and mesh configuration. (**a**) Simulation box consists of a rectangular duct with the cross-sectional dimensions W = 50 µm, H = 25 µm and length of L_C_ = 150 µm; channel walls move with the speed of −U_p_ while the particle has angular velocity vector of Ω. (**b**) Domain mesh (inlet and interior mesh are hidden for easier visualization) (**c**) cross-section mesh. Y_P_ and Z_P_ are the distance of the particle from the side wall and the bottom wall, respectively. (**d**) Finer mesh on the particle surface and boundary layer mesh surrounding the particle.

Full 3-D incompressible Navier–Stokes equations are solved in the steady state in order to quantify flow variables. The simulation domain consists of a 25 µm × 50 µm (H × W) rectangular duct with the length of L_C_ = 150 µm as a segment of the microchannel and a spherical hole with diameter d is embedded inside the domain representing the particle (Figure 2a). *Viscoelastic Flow* interface (*vef*) in COMSOL Multiphysics 5.6^®^ (COMSOL, Inc., Stockholm, Sweden) is used to solve the momentum and mass conservation equations which can be written as:

(1) $$\rho \left(u\cdot \nabla \right)u=\nabla \cdot \left(-pI+\mu_{s}\left(L+L^{T}\right)+T_{e}\right)$$

(2) ∇·u=0

where $\rho ,\mu_{s},\;\mathrm{and}\;I$ are the fluid density, solvent viscosity, and identity matrix, respectively, and ***T_e_*** is the extra elastic stress tensor. ***L*** is the velocity gradient tensor. Fluid density and solvent viscosity is set to 1000 kg/m^3^ and 1 mPa·s, respectively. Periodic flow condition with appropriate pressure difference values is used to generate the flow inside the channel, and quadratic and linear shape functions are chosen for the discretization of the velocity and pressure fields, respectively. Translational velocity (U_P_) of the particle in the downstream direction is modeled by applying velocity of −U_P_ to the channel walls, while rotation is simulated using appropriate moving wall boundary condition on the particle surface to account for the rotational velocity—$\phi_{x},\;\phi_{y},\;\mathrm{and}\;\phi_{z}$—about all axis.

#### 2.2.2. Constraint Particle Rotational and Angular Velocities

It is important to note that the calculation of lift force is accurate only in the case of a neutrally buoyant particle, meaning that in the steady state, force in the flow direction, and torque about all axis on the particle must be zero [40]. Therefore, the Navier–Stokes equations are coupled with appropriate global equations in order to constraint particle translational (Equation (3)) and angular velocities (Equation (4)) to maintain the aforementioned conditions. These equations are given as:

(3) $$∯\left[-pI+\mu_{s}\left(L+L^{T}\right)+T_{e}\;\right]\;n\;dS=0$$

(4) $$∯r\times \left\{\;\left[-pI+\mu_{s}\left(L+L^{T}\right)+T_{e}\right]\;n\;\right\}\;dS=0$$

where Equation (3) is calculated in the X-direction to impose zero net force on the particle at steady state in the flow direction and Equation (4) is computed in all directions to ensure that the particle is rotating torque-free. The integrals are calculated over the particle surface. Lateral forces on the particle are then obtained by integrating total stress in the Y and Z directions. We can also decouple inertial and elastic components of the lift force by probing each one of the contributions separately (integrating the extra elastic stress ($T_{e}$) over the particle surface yields the elastic force; integrating pressure and the inertial stress gives the inertial component of the lift force). Note that due to symmetry, force values are calculated only along half of each centerline; therefore, Y and Z-midlines are referred to the distance between the channel wall and the center along the Y and Z directions, respectively (Figure 2a).

#### 2.2.3. Mesh Dependence and Model Validation

Simulation domain consists of about 15 × 10^4^ tetrahedral mesh elements (Figure 2b) with two levels of refinement to ensure accuracy of the results. Extra fine triangular mesh with the average element size of 0.1 d is employed on the particle surface and four stretching boundary layer mesh are used around the particle. This guarantees capturing of sharp stress and velocity gradients in the vicinity of the particle (Figure 2c,d). They specifically help with the convergence of the model at high *Wi* numbers where these sharp gradients are expected. Equations solved under this mesh configuration will generate about 85 × 10^4^ degrees of freedom which meets our computational capacity when solving multiple cases in parallel as an attempt to optimize computational time while maintaining sufficient accuracy. The simulation scheme used here was previously validated by Di Carlo et al. [40]. Independence of the simulation results on the number of mesh elements is shown in Figure S1.

#### 2.2.4. Giesekus Constitutive Equation

**Figure 3:**
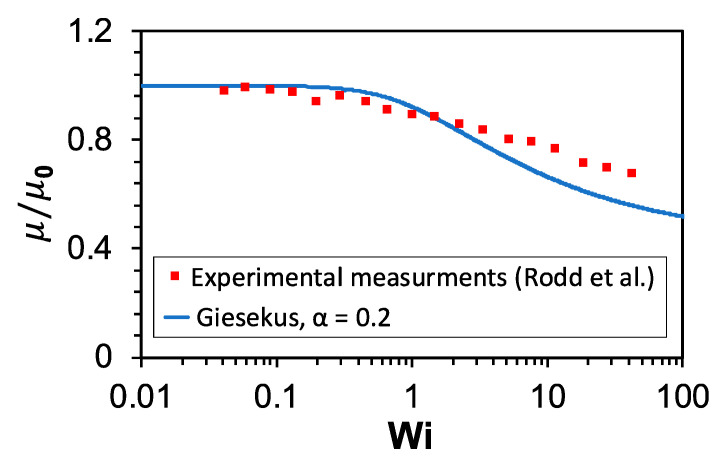
Shear-thinning behavior of the 1000 ppm PEO solution. Comparison of the normalized viscosity as a function of *Wi* number between experimental measurements by Rodd et al. [58] and the *Giesekus* model with the mobility factor α = 0.2.

Due to the shear-thinning behavior of the PEO solution [46,57,58], *Giesekus* constitutive equation [59] is used to model the viscoelasticity of the fluid and to calculate the extra elastic stress tensor. In steady state, *Giesekus* model is given as

(5) $$\lambda \overset{\nabla }{T_{e}}+\left(1+\frac{\alpha \lambda }{\mu_{p}}T_{e}\right)T_{e}=2\mu_{p}D$$

(6) $$\overset{\nabla }{T_{e}}=\left(u\cdot \nabla \right)T_{e}-L\cdot T_{e}-T_{e}\cdot L^{T}$$

where ***D*** is the strain rate tensor and is defined as $\frac{1}{2}\left(L+L^{T}\right)$. Equation (6) is the upper convective derivative of the elastic stress tensor in the steady state. $\lambda \;\mathrm{and}\;\mu_{p}$ are the relaxation time and polymer part of the viscosity of the fluid and are set to 6.8 ms and 1.3 mPa·s, respectively, and α is the mobility factor. The total viscosity of the fluid is defined as $\mu =\;\mu_{s}\;+\;\mu_{p}.$ Note that the extent of shear-thinning behavior of the fluid can be fine-tuned by adjusting the α constant in the *Giesekus* model. Based on experimental measurements by Rodd et al. [58], the normalized viscosities ($\mu /\mu_{0}$) of 1000 ppm PEO solution at the working conditions of our experiments, *Wi* = 3.6, and 18, are 0.83 and 0.71, respectively, where $\mu_{0}$ is the initial total viscosity at zero shear rate. This shear-thinning behavior can be reasonably estimated by setting α=0.2 in the *Giesekus* equation (Figure 3). Therefore, this value of the mobility factor will be used in our simulations to capture the shear-thinning behavior of the viscoelastic fluid. The shear-rate dependent viscosity of the PEO solution might also be modeled using the fractal derivative rheological model recently introduced by Zuo and Liu [60]. This model can correctly predict the non-linear velocity distribution of SiC paste.

## 3. Results and Discussion

### 3.1. Experimental Results

**Figure 4:**
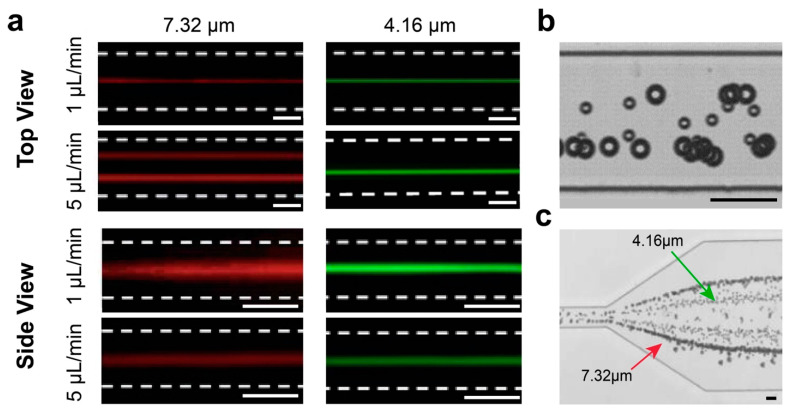
Experimental observations of focusing and separation of the 4.16 µm and 7.32 µm diameter particles in viscoelastic flow. (**a**) Fluorescent streak images of top and side views at 1 µL/min (*Wi* = 3.6) and 5 µL/min (*Wi* = 18) in 50 µm × 25 µm microchannel. (**b**) Top view bright-field image confirming distinct focusing positions of the two particle sizes at Q = 5 µL/min. (**c**) Expansion region at the channel outlet enabling separation of the focused streams. (Scale bar in all images: 25 µm).

Using top view and side view imaging methods, we found that particles were vertically focused into the middle plane, while focusing pattern can vary laterally in microfluidic rectangular channels depending on particle size and flow condition. Fluorescent and bright-field images were used to investigate the focusing behavior of particles in the PEO solution at 1 µL/min (*Re* = 0.2, *Wi* = 3.6, *El* = 18) and 5 µL/min (*Re* = 1, *Wi* = 18, *El* = 18). At low flowrate (Q = 1 µL/min), both the 4.16 µm and the 7.32 µm particles were laterally focused in the center of the channel width as shown in Figure 4a. Increasing the flowrate to Q = 5 µL/min, the single focusing position in the center was bisected into two streams for both particle sizes. On the other hand, both the 4.16 µm and the 7.32 µm particles were focused vertically in the middle of the channel height at both flowrates (Figure 4a). Such observations suggest a single focusing position at low flowrate in the center of the cross section and two focusing positions near the sidewalls at higher flowrate. Thus, 3D focusing of particles can be achieved for the 4.16 µm and the 7.32 particles at 1 µL/min, which is particularly desirable for flow cytometry applications. [44] Additionally, line scan data showed that at 5 µL/min, the 4.16 µm particles focus ≈ 4 µm closer to the channel center, compared to the 7.32 µm particles (Experimental line scan data for all cases are presented in Figure S2). This is further confirmed in the top view bright field image of the binary particle mixture at 5 µL/min (Figure 4b). As a result, separation of the particles can be achieved using an expansion region at the outlet of the channel (Figure 4c).

The three main observations from the experiments can be summarized as follows: (1) both particle sizes focused in a single stream at the channel center at low flowrate, (2) particles moved away from the center along the Y-midline of the channel at higher flowrate, and (3) large particles are focused further away from the channel center at high flowrate, which enabled particle separation based on size. Similar behavior was previously reported by Li et al. [48]. They experimentally observed that 3, 5, and 10 µm particles focus in distinct positions in the Y-midline of a rectangular channel (*Re* = 0.97, *Wi* = 18.1), with the 3 µm particle spreading around the centerline, 5 µm particle splitting into two streams and focus at a distance of 1/3 channel width away from the center, and the 10 µm particle focus even further away from the center, at a distance of 2/3 channel width. Therefore, based on the focusing trends, the equilibrium positions tend to shift away from the channel centerline and towards the walls by increasing the flowrate and also, by increasing the particle size.

Next, we used simulation results to investigate the underlying physics behind such behavior and propose general mechanisms that govern particle lateral migration at this range of flowrate.

### 3.2. Simulation Results

#### 3.2.1. Force Analysis along Y-Midline

**Figure 5:**
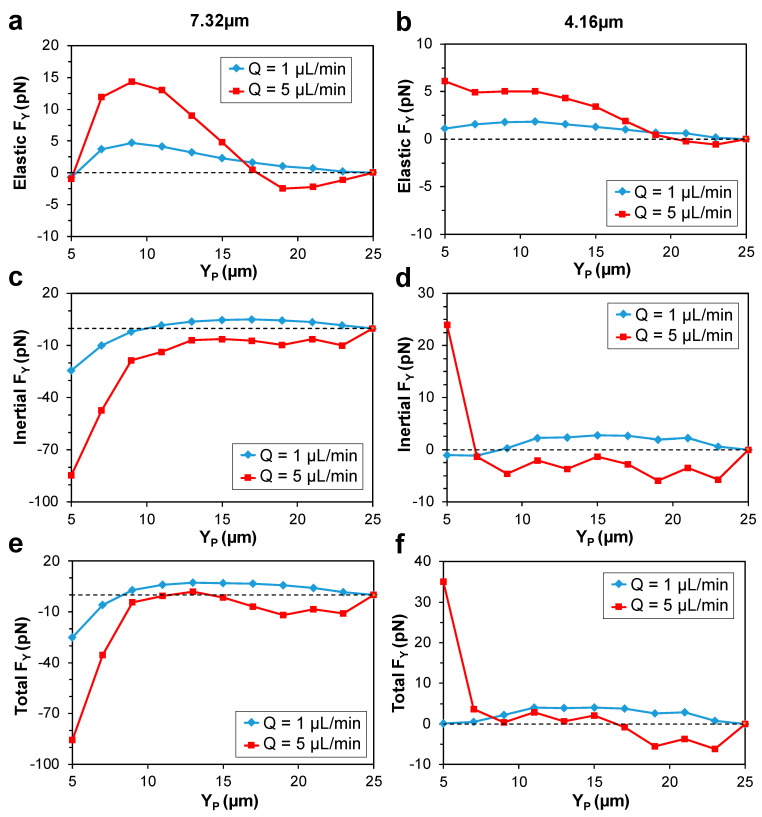
Horizontal force (F_Y_) distribution along the Y-midline of the channel. Elastic force profile for the (**a**) 7.32 µm and (**b**) 4.16 µm diameter particles at *Q* = 1 µL/min (*Wi* = 3.6) and *Q* = 5 µL/min (*Wi* = 18). Inertial force profiles for the (**c**) 7.32 µm and (**d**) 4.16 µm diameter particles at the two flowrates. Total force profiles for the (**e**) 7.32 µm and (**f**) 4.16 µm diameter particles at the two flowrates.

To find the stable and unstable equilibrium positions of particles and understand distinct effects of inertia and elasticity on the focusing patterns, horizontal force values were plotted along the Y-midline of the channel. Force curves for low (1 µL/min) and high (5 µL/min) flowrates are presented in Figure 5. Positive and negative force values correspond to the center-directed and wall-directed forces, respectively. Therefore, a stable focusing position is located on the Y-midline where the force curve crosses the F = 0 line with a negative slope. This indicates a converging point for the forces where the particle will be ‘trapped’ and in turn, focused. Conversely, unstable equilibrium positions are the locations on the Y-midline where the force curve intersects the F = 0 line with a positive slope, i.e., a diverging point. That is, although the force value is zero, any disturbance to the particle will lead to defocusing. The same is true, for the Z-midline force plots. F = 0 lines are shown on the plots with a dashed line. Simulated data are presented along half the centerlines due to symmetry. Although F_SFD_ is caused by the N_2_-induced secondary flow which is a viscoelastic phenomenon, it will impact the non-elastic stress component due to the inertial nature of the drag force. Therefore, fluid viscoelasticity will impact particle migration directly due to F_E_, and indirectly through F_SFD_.

Generally, a center-directed elastic force is expected to push the particles towards the lowest first normal stress difference (N_1_) regions [61], with the maximum force magnitudes occurring closer to the channel walls where stronger N_1_ is present, as seen in Figure S3. This behavior of the elastic force was obtained at Q = 1 µL/min (*Wi* = 3.6) for both particle sizes and is presented in Figure 5a,b. On the other hand, at Q = 5 µL/min (*Wi* = 18), unexpected negative elastic force values were obtained near the channel center. The wall-directed migration of particles at high flowrates is typically attributed to the shear-thinning effects only. While this new finding suggests that the migration of particles away from the channel center at higher flowrates can also be due to the direct effect of the elastic force. This counter-intuitive result can be explained by the possible disturbance caused to the N_1_ distribution on the surface and at the vicinity of the particle at higher *Wi* numbers.

Inertia-driven migration of particles in *Newtonian* fluids is mainly attributed to the presence of a center-directed wall-induced force, F_W,_ and a wall-directed shear-gradient force, F_S_. In viscoelastic fluids however, addition of F_SFD_ and shear-thinning effects can impact the traditional inertial force behavior. The inertial force distribution for the 7.32 µm particle at the Y-midline is shown in Figure 5c. At Q = 1 µL/min, inertial force was positive along the whole midline except for the near the wall region (Y_P_ < 10 µm). The positive force at the central region could be caused by the presence of the relatively strong center-directed N_2_-induced secondary flow along the Y-midline of the channel. The formation of secondary flow in the cross-section of the channel is shown in Figure S4. Conceivably, shear-gradient lift (F_S_) is simply not strong enough at this flowrate to counteract the transversal drag force caused by the secondary flow (F_SFD_). Conversely, at the near the wall region, negative inertial force values were obtained. This behavior is in agreement with the observations by Raffiee et al. [54] near the wall for *Giesekus* fluid, and in contrast with the expected behavior of inertial force near the wall in *Newtonian* and non-shear-thinning fluids such as *Oldroyd-B* fluid, where positive, wall-repulsive force (F_W_) is expected to push the particles away from the wall [39,41,51].

**Figure 6:**
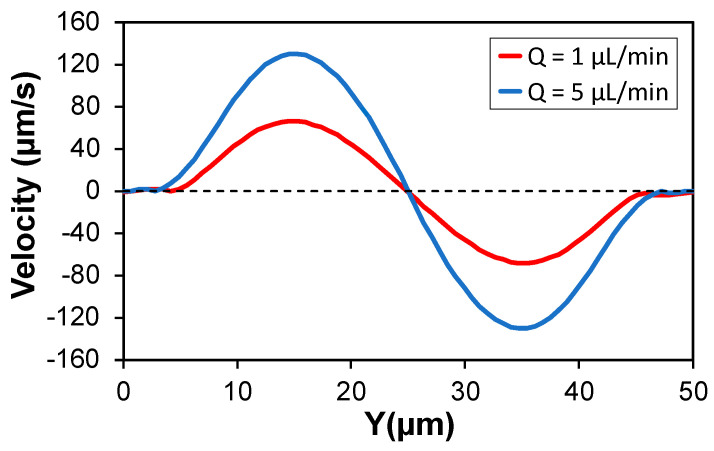
Effects of flowrate on the magnitude of N_2_-induced secondary flow orthogonal to the main flow direction along the channel horizontal centerline. Magnitude of the secondary flow is almost doubled when increasing the flowrate from 1 µL/min (*Wi* = 3.6) to 5 µL/min (*Wi* = 18) at constant α = 0.2.

To more accurately quantify the effects of F_SFD_ on the net inertial force distribution, we plotted the secondary flow magnitude as a function of flowrate (Figure 6). It is observed that the magnitude of the secondary flow is weakly proportional to the flowrate, as the maximum velocity was only doubled when increasing the flowrate from 1 µL/min to 5 µL/min. The N_2_-induced transversal drag force can be approximated by the Stokes’ drag formulation [62], where the force is linearly proportional to the velocity of the fluid. Therefore, F_SFD_ is weakly proportional to the increase in flowrate. At Q = 5 µL/min, inertial force was negative for the 7.32 µm particle in the whole midline. The magnitude of the negative F_S_, is proportional to the second power of the flowrate [63]. As a result, F_S_ overcomes F_SFD_ at high flowrate and generates negative net inertial force. Conversely, a different behavior was obtained for the 4.16 µm particle. At Q = 5 µL/min, inertial force took positive values near the wall and negative values near the center (Figure 5d). This behavior is identical to the inertial force distribution on a particle in *Newtonian* fluid.

Superposition of the inertial and elastic force curves along the Y-midline yielded the total force. For the 7.32 µm particle, at 1 µL/min, positive force pushes the particles all the way towards the center of the channel where the particles are focused (Figure 5e). This is in agreement with our experimental observations for the 7.32 µm particle at this flowrate, where the fluorescence intensity is peaked at Y = 25 µm, i.e., channel center, from the top view (Figure S2). Additionally, an unstable focusing position was obtained at 8 µm away from the wall and the particles are expected to be pushed towards the wall if their initial position is Y_P_ < 8 µm. Under this strong wall-directed force, the particle is either trapped in the proximity of the wall, or hits the wall and bounces back toward the center. Therefore, we term it the “attraction-repulsion” region.

When increasing the flowrate, the center focusing position shifted away from the center and moved closer to the sidewalls. Consequently, due to symmetry, two focusing bands are predicted to form on the horizontal centerline of the channel, which was also confirmed in the experiments. In our experiments, line scan data at 5 µL/min showed that for the 7.32 µm particle, intensity of the fluorescence top view images peaks at Y ≈ 15 µm and Y ≈ 35 µm (Figure S2). In the simulations, the stable focusing position was obtained at Y_P_ ≈ 14 µm (also Y_P_ ≈ 36, due to symmetry). Therefore, simulations predict the experimental focusing positions of the 7.32 µm particle almost perfectly at both 1 µL/min and 5 µL/min. The attraction-repulsion region near the wall exists at the high flowrate as well.

The 4.16 µm particle exhibited no attraction-repulsion region near channel walls at neither of the flowrates. The total force exerted on the 4.16 µm particle is plotted in Figure 5f. At Q = 1 µL/min, particles experience positive force all the way towards the channel center. By increasing the flowrate to Q = 5 µL/min, negative force near the center shifted the equilibrium position away from the center, leading to particle focusing at Y_p_ ≈ 17 µm (also Y_P_ ≈ 33 µm, due to symmetry). Experimental line scan data for the 4.16 µm particle at 5 µL/min peaked at Y ≈ 29 µm (due to the non-uniform input, we only captured one stream near the bottom sidewall) (Figure S2). Therefore, simulation results perfectly predict the focusing position of the 4.16 µm particles at 1 µL/min. However, the predicted value of the equilibrium position at 5 µL/min is ≈ 4 µm different from that observed in the experiments. This could potentially be due to the channel not being long enough for the 4.16 µm particles to complete their migration towards the stable focusing positions.

Based on our results, focusing position of particles moved away from the channel center by increasing the flowrate. This can be attributed to the negative shear-gradient lift dominating particle migration in the central regions, specifically since there is no opposing positive elastic force to counteract it in the central regions at high flowrate. In fact, as discussed earlier, the elastic force can promote the wall-directed migration at Q = 5 µL/min. Same behavior was previously observed in *Newtonian* fluid. Amini et al. [64] showed that by increasing *Re*, focusing position of particles in square and rectangular microchannels shift slightly towards the channel walls. On the other hand, effect of particle size is rather complex. In pure inertial flow, it is expected that the focusing position shift towards the channel centerline as the confinement ratio (d/H) increases [40]. For our case of elasto-inertial focusing, at Q = 5 µL/min, the inertial force took negative values for both particle sizes in the whole midline except for the 4.16 µm particle near the channel walls. In fact, this is the opposite of the observation reported for the confinement ratio effect in pure inertial flow mentioned above, since the larger particle size is now pushed more closely to the channel walls at higher flowrate. Elastic force however, becomes dominant in the intermediate region between the channel center and the walls, counteracting the negative net inertial force. This creates a diverging point for the total force, and hence, leads to the focusing of particles. This crucial contribution of the elastic force is what makes the phenomenon called *elasto-inertial* focusing, otherwise it would only be a special case of inertial focusing in a shear-thinning fluid as in the studies by Hu et al. [65] and Chrit et al. [66] where inertial migration of particles in channel flows of power law fluid is investigated, without the presence of elastic force.

#### 3.2.2. Force Analysis along Z-Midline

**Figure 7:**
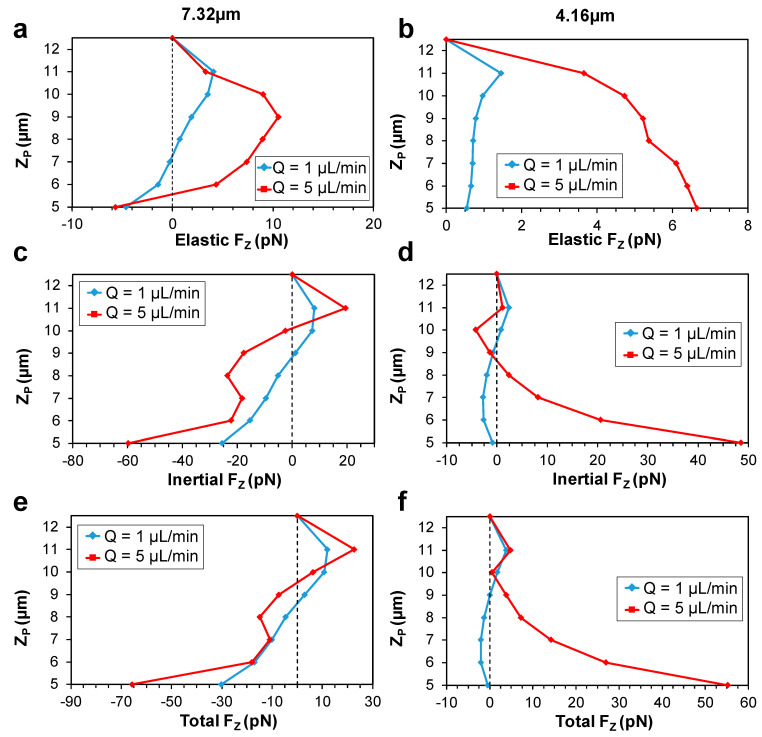
Vertical force (F_Z_) distribution along the Z-midline of the channel. Elastic force profile for the (**a**) 7.32 µm and (**b**) 4.16 µm diameter particles at *Q* = 1 µL/min (*Wi* = 3.6) and *Q* = 5 µL/min (*Wi* = 18). Inertial force profiles for the (**c**) 7.32 µm and (**d**) 4.16 µm diameter particles at the two flowrates. Total force profiles for the (**e**) 7.32 µm and (**f**) 4.16 µm diameter particles at the two flowrates.

Next, we plotted Z-midline force curves and analyzed the obtained results by comparing with the side view experimental images. The elastic force follows almost identical patterns as the Y-midline elastic force curves, except for the 7.32 µm particle near the channel walls (Figure 7a,b). This somewhat counter-intuitive behavior for the elastic force was also observed previously by Raffiee et al. [54] at a range of lower *Wi* numbers (*Re* = 5, *Wi* = 0.5, and also *Re* = 10 and *Wi* = 0.1, 0.5, and 3) on a particle with blockage ratio k = 0.3, in a shear-thinning *Giesekus* fluid in a square channel. Note that in the current study, the blockage ratio of the 7.32 µm particle with respect to the Z-midline is calculated as d/H = $\frac{7.32}{25}$ = 0.29, which is relatively close to the blockage ratio in Raffiee’s report.

The inertial force curves showed that for the 7.32 µm particle, the inertial force was negative at Z_P_ < 9, but changed sign and became positive near the center (Figure 7c). For the 4.16 µm particle, inertial force behaved similarly near the wall as in the Y-midline and exerted a positive, center-directed force to the particles. The force curves then started to change sign and took negative values in the intermediate region between the channel walls and the channel center. However, similar to the 7.32 µm particles, they experienced a positive force in the proximity of the channel centerline (Figure 7d). This positive trend near the center for both particle sizes was only observed in the Z-midline. However, it cannot be attributed to the difference in the blockage ratio of particles in the two midlines. The Z-midline blockage ratio of the 4.16 µm particle, $\frac{4.16}{25}$ = 0.16, is almost equal to the Y-midline blockage ratio of the 7.32 µm particle, $\frac{7.32}{50}$ = 0.15. However, the positive trend was not observed for the 7.32 µm particle in the Y-midline.

Lastly, the total force curves along the Z-midline are analyzed. For the 7.32 µm particle, an unstable equilibrium position occurred at 8.5 < Z_P_ < 9.5 for both flowrates, where due to any minimal disturbance, the particles were either pushed towards a stable equilibrium position in the vertical centerline of the channel (Z_P_ = 12.5), or to an attraction-repulsion region near the channel walls. However, for the 4.16 µm particle, the attraction-repulsion region was only observed at Q = 1 µL/min. At the high flowrate (Q = 5 µL/min), only one, stable focusing position was obtained at the vertical centerline of the channel. The center stable focusing positions obtained in the simulations are in agreement with the experimental observations. The line scan data showed that the fluorescent intensity peaks at the channel vertical center (Z = 12.5 µm) for both particle sizes at 1 µL/min and 5 µL/min (Figure S2).

A schematic of the comparison between the experimental observations and simulation results for the focusing position of both particle sizes is presented in Figure S5. The cross-sectional picture is based on the top and side view images and the Y-midline and Z-midline force curves.

### 3.3. General Focusing Mechanisms

Based on our proposed mechanisms, the shear-gradient (F_S_), N_2_-induced secondary transversal drag (F_SFD_), and the elastic (F_E_) forces balance particles laterally, leading to their final, stable, equilibrium positions. These conclusions are based on the Y-midline force curves. Wall-induced force (F_W_) has little to no effect on the focusing mechanisms because the focusing occurs considerably far from the channel walls in all cases.

**Figure 8:**
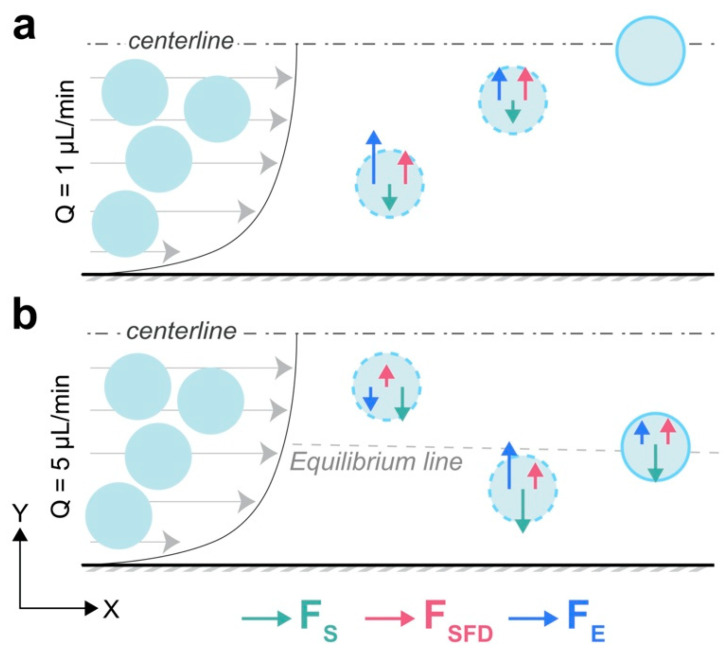
General focusing mechanisms of particles in the Y-midline of a rectangular channel in viscoelastic fluid flow. Final stable equilibrium position of particles is determined by the balance of the shear-gradient (F_S_), N_2_-induced secondary flow transversal drag (F_SFD_), and the elastic (F_E_) forces at (**a**) *Q* = 1 µL/min (*Wi* = 3.6), and (**b**) *Q* = 5 µL/min (*Wi* = 18). Dashed circles indicate particles migrating towards the equilibrium position; solid circles are particles at final, stable equilibrium position.

At Q = 1 µL/min, particles experienced negative F_S_ along the whole midline, which was not strong enough to overcome the positive F_SFD_, and therefore, net inertial lift force remained positive as seen in the Y-midline inertial force curves (Figure 5c,d)**.** This positive net inertial lift, alongside with the positive F_E_ pushed the particles towards the center of the channel (Figure 8a). At Q = 5 µL/min, particles near the center experienced negative elastic, and net inertial lift forces and hence moved away from the center. This wall-directed migration persisted until the strong positive F_E_ near the walls counteracted negative net inertial force and pushed back the particles towards the equilibrium line where negative F_S_ counterbalanced positive F_E_ + F_SFD_ (Figure 8b). This mechanism can also explain the reason behind the 4.16 µm particles focusing closer to the channel center compared to the 7.32 µm particles at Q = 5 µL/min. Based on the above conclusion, decreasing particle size decreases the net positive lift force (F_SFD_ + F_E_) more significantly than the negative component of the lift (F_S_). This occurs because both F_E_ and F_S_ scale with ∼d^3^ [41,61], while F_SFD_ which can be simply derived by the stokes drag formulation scales with ∼d. As a result, the 4.16 µm particle is focused closer to the channel center.

**Figure 9:**
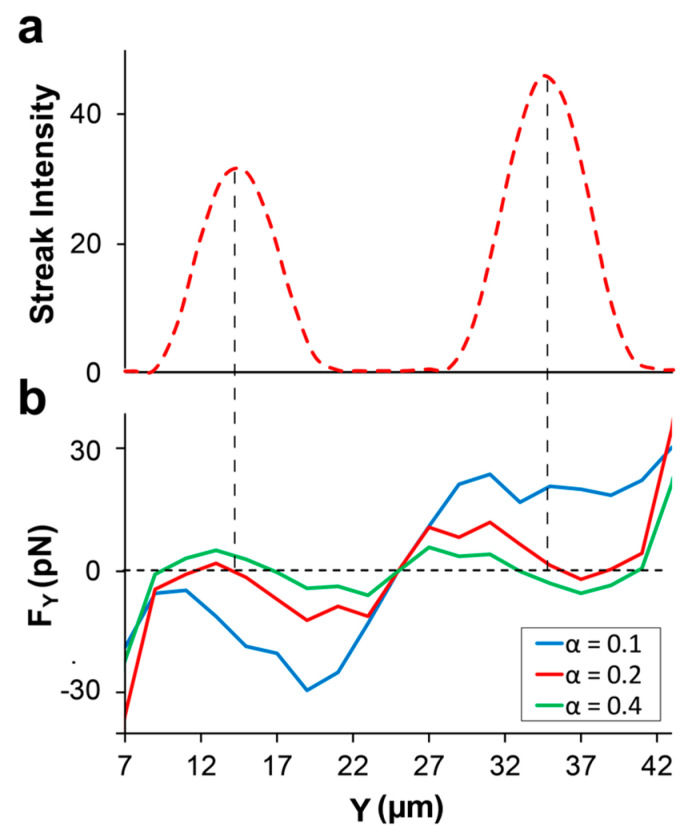
Effects of fluid shear-thinning on the prediction of focusing position. (**a**) Experimental results for the streak intensity of the 7.32 µm diameter particles in 1000 ppm PEO at *Q* = 5 µL/min (*Wi* = 18). (**b**) Simulation results for three different alpha constants. The α = 0.2 predicts the focusing positions precisely, while overestimating shear-thinning extent of the fluid (α = 0.4) predicts focusing positions closer to the channel center, and underestimating alpha (α = 0.1) predicts no focusing in the central regions.

**Figure 10:**
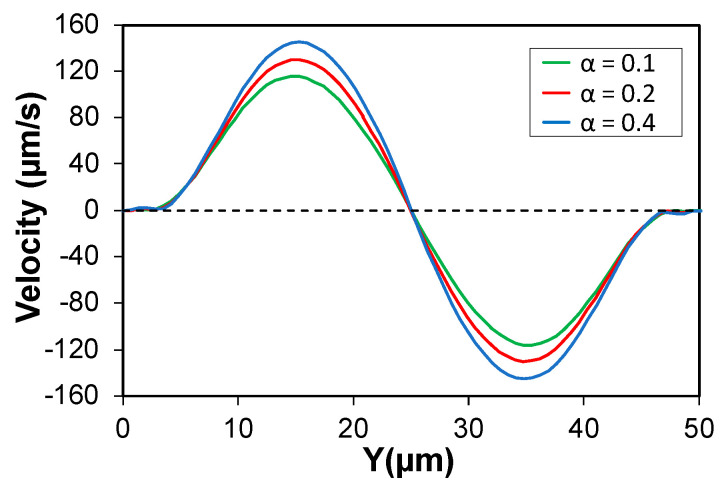
Effect of the mobility factor (α) on the magnitude of the N_2_-induced secondary flow. Higher α values increase the magnitude of the secondary flow orthogonal to the main flow direction along the channel horizontal centerline.

The correct and precise prediction of the focusing positions was possible when rheological properties of the carrying medium were the closest to their actual values. For instance, the predicted focusing position of the 7.32 µm particle at Q = 5 µL/min was strongly related to value of the mobility factor (α) in the *Giesekus* equation. The streak intensity plot for the 7.32 µm particle at 5 µL/min is shown in Figure 9a and the simulation results with three different mobility factors of α = 0.1, 0.2, and 0.4 are plotted in Figure 9b. When α = 0.2, simulations predict the focusing position almost perfectly. However, as expected based on our mechanism, the predicted focusing position shifted towards the channel center when α = 0.4. This center-directed shift happened since (1) F_S_ was weakened due to the more flattened velocity profile at higher alpha, and (2) F_SFD_ got stronger with increasing α. The relationship between alpha and secondary flow magnitude, which is linearly proportional to F_SFD_, is illustrated in Figure 10 and is in agreement with observations by Villone et al. [53]. Conversely, when α = 0.1, stronger F_S_ overcomes opposing F_SFD_ (now weakened) and pushes the particle all the way to the channel walls, leading to no stable focusing position along the Y-midline. If D_C_ is the distance of the particle measured from the channel center, the error percentage for the predicted focusing position can be defined as: E = $\frac{|\;(D_{C})\;simulation-(D_{C})\;experiment\;|\;}{(D_{C})\;experiment}\times 100$. When α = 0.2, average E for the two focused bands was ≈ 4.15%. However, in the case of overestimation of the mobility factor, α = 0.4, average E ≈ 21.3%. On a side note, it is worth mentioning that the behavior of particles in a rectangular channel flow of viscoelastic fluid can be different from their behavior in a square channel. In a square channel, eight vortices are formed in the cross-section of the channel [53], pushing the particles towards the channel walls in all orthogonal directions, while direction of the secondary flow is towards the channel center on the Y-midline of a rectangular channel. Therefore, F_SFD_ and F_S_ might compete in some cases, while they always have synergetic effects in square channels.

Lastly, the fact that there were no particles observed near the channel walls in the experiments could be due to a number of possibilities: (1) Only a small percentage of particles’ initial positions were at the regions that promoted near the wall focusing position, (2) The attraction-repulsion region was in fact more of a repulsion region where particles were bounced back after hitting the wall. (3) Particles, although attracted towards the wall horizontally, were not vertically balanced, i.e., these attraction basins were unstable focusing positions in the Z-direction, leading to particles moving up or downwards due to minimal disturbance, and (4) The particles near the channel walls may be rare events compared to the focusing of particles in major positions as shown in Figure 4. Capturing such rare events can be very difficult in our current imaging setting. Additionally, the fluorescent signal of these rare events can be too weak to be detected in the stacked images even if they were successfully captured.

### 3.4. Shear-Thinning Effects near the Channel Walls

It is known that the wall-induced lift (F_W_) is a center-directed force generated due to the build-up of pressure in the narrow region between the particle and the channel walls if the particle is located close enough to the wall [26]. Conversely, shear-gradient lift (F_S_), is a wall-directed force due to the parabolic nature of the velocity profile inside the channel. This parabolic velocity distribution will cause the particle to experience different relative velocities on the two sides that are facing the wall (V_W_) and the channel center (V_C_), and this will generate a force towards the channel walls in order to minimize this velocity imbalance [26].

**Figure 11:**
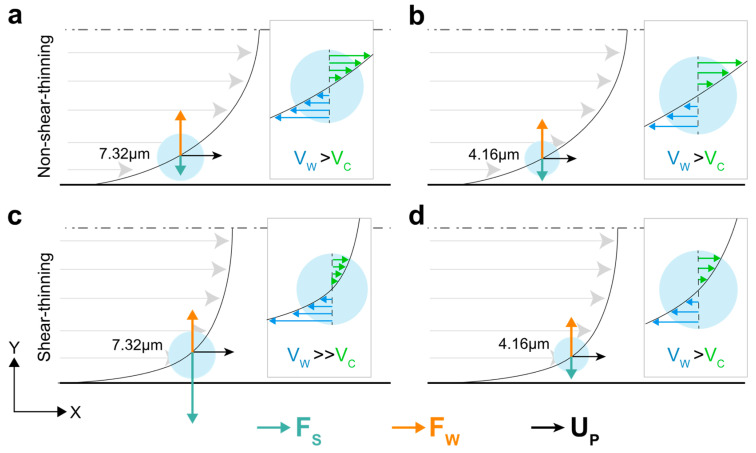
Schematics of the effect of shear-thinning behavior of the fluid on the inertial force near the channel walls. Both (**a**) 7.32 µm and (**b**) 4.16 µm diameter particles experience positive net inertial force (F_W_ > F_S_) in non-shear-thinning fluids. (**c**) Shear-thinning promotes the shear-gradient lift force (Fs) by imposing stronger relative velocity imbalance on the particle surface for the 7.32 µm particles, causing net inertial force to be negative near the channel walls. (**d**) The 4.16 µm diameter particles are not large enough to capture this relative velocity imbalance.

Schematics of the relative magnitude and direction of wall-induced and shear-gradient lift on both particle sizes in a non-shear-thinning fluid is illustrated in Figure 11a,b. Since wall-induced is the dominant contributor of the two components, particles experience a repulsive force when located significantly close to the channel walls. In the case of a shear-thinning fluid however, formation of the warped velocity profile, will impact the shear-gradient force on the two particle sizes by manipulating relative velocities on the particle surface (Figure 11c,d). For the 7.32 µm particle, V_W_ is significantly larger than V_S_, creating an amplified shear-gradient force, which is strong enough to overcome the wall-induced force and in turn, net inertial force pushes the particle towards the channel walls. The 4.16 µm particle however, will not experience this enhanced shear-gradient due to its smaller size, and hence, wall-repulsive force is still the dominant of the two. As a result, net inertial force exerted on the smaller-sized particle will be, at least qualitatively, similar to that in a non-shear-thinning fluid. This behavior was also previously observed by D’Avino et al. [50] in a shear-thinning flow of particles in a cylindrical micropipe. They observed that by increasing the blockage ratio, particles tend to focus near tube walls rather than the centerline. Conversely, inertial force behavior observed in an *Oldryod-B* fluid is identical to that of the *Newtonian* fluid, as in the study by Raoufi et al. [51], where only center-directed inertial force is observed near the channel walls. This can further support the idea that the strong wall-directed inertial force near the walls in a *Giesekus* fluid is indeed due to the impact of the shear-thinning on the velocity profile.

## 4. Conclusions

In this work, we described a general mechanism for the focusing of particles in the *elasto-inertial* regime in a straight, rectangular microchannel. 3-D direct numerical simulations as well as fluorescent and bright-field experimental images were used to accurately quantify the focusing and separation of 4.16 µm and 7.32 µm diameter particles in a rectangular channel flow of *Giesekus* fluid at low (1 µL/min) and high (5 µL/min) flowrates corresponding to *Wi* = 3.6 and *Wi* = 18, respectively. Our results revealed that the elastic force (F_E_) and N_2_-induced secondary flow transversal drag (F_SFD_) push both particle sizes towards the channel center at low flowrate (*Wi* = 3.6). At high flowrate (*Wi* = 18), two focusing positions are observed on the Y-midline of the channel due to the balance of F_E_, F_SFD_, and shear-gradient lift force (F_S_). We reported, for the first time, that elastic force can promote wall-directed migration of particles in the central regions at *Wi* **~** O(10). Due to the different scaling of the forces responsible for the focusing with the particle diameter, smaller particles equilibrate closer to the channel center. Additionally, we demonstrated that the correct prediction of the focusing positions is directly associated with the correct estimation of the mobility factor or the α constant in the *Giesekus* equation. Over or underestimation of the shear-thinning extent will lead to substantial error in the predicted focusing positions that can negatively affect the efficiency of particle separation devices. Lastly, we showed that the unique behavior of the inertial force near the channel walls can be explained by exploring the impact of the shear-thinning property of the fluid on the velocity profile. The ‘warped’ velocity gradient present in shear-thinning fluid amplifies the shear-gradient force. This amplified force will overcome the wall-induced lift, causing the net inertial force to be towards the walls in such fluids.

## Figures and Tables

**Table 1:** Selected existing studies on the focusing and separation of particles in viscoelastic fluid.

Reference	Approach		Fluid Type Used in Exp.	Flow Regime	Geometry	Considered in Simulations	
	Exp.	Sim.				Shear-Thinning	Secondary Flow
Li et al., (2016) [48]	✓	✕	PVP, PAA, PEO	0.1 < *Re* < 2 0 < *Wi* < 30	Rectangular channel	-	-
Li and Xuan (2019) [49]	✓	✕	XG	0.01 < *Re* < 30	Rectangular channel	-	-
Feng et al., (2019) [45]	✓	✕	PEO	10^−4^ < *Re* < 10^2^ 10^−3^ < *Wi* < 10^3^	Rectangular spiral channel	-	-
Di’Avino et al., (2012) [50]	✓	✓	PVP, PEO	Inertia-less *De* ^i^ < 2	Circular tube	Yes	Yes
Raoufi et al., (2019) [51]	✓	✓	PEO	0 < *Re* < 20 ^ii^ 1 < *Wi* < 220 ^ii^	Square, rectangular, trapezoidal and complex channel	No	No
Huang & Joseph (2000) [52]	✕	✓	-	0 < *Re* < 56 *De* < 2.6	2-D parallel walls	Yes	No
Villone et al., (2013) [53]	✕	✓	-	Inertia-less *De* < 6	Square channel	Yes	Yes
Raffiee et al., (2019) [54]	✕	✓	-	*Re* < 30 *Wi* < 3	Square channel	Yes	Yes
Wang et al., (2018) [55]	✕	✓	-	*Re* = 1 & 50 *Wi* < 2.5	Rectangular channel	Yes	Yes
Present study	✓	✓	PEO	*Re* = 0.2 & 1 *Wi* = 3.6 & 18	Rectangular channel	Yes	Yes

^i^ *De* is the Deborah number, calculated as *De* = $\frac{\lambda }{t_{p}}$ where $t_{p}$ is the characteristic time scale. ^ii^ *Re* and *Wi* ranges are calculated based on the given information in the paper.

## Data Availability

The data that support the findings of this study are available within this article.

## Supplementary Materials

The following supporting information can be downloaded at: https://www.mdpi.com/article/10.3390/mi13122131/s1, Figure S1: Mesh independence test results; Figure S2. Line scan data of the fluorescent intensity of the focused streams for the 4.16 μm and 7.32 μm particles at (a,b) 1 μL/min, top view, (c,d) 5 μL/min, top view, (e,f) 1 μL/min, side view, (g,h) 5 μL/min, side view; Figure S3. Simulation result of the first normal stress difference (N1) at Q = 1 μL/min. (Re = 0.2, Wi = 3.6, El = 18); Figure S4. Simulation result of the magnitude and direction of the N2-induced secondary flow at Q = 1 μL/min. (Re = 0.2, Wi = 3.6, El = 18); Figure S5. Channel cross-section schematic comparing the experimental (colored circles) observations and the simulation results (dashed circles) for the focusing position of 4.16 μm and 7.32 μm particles at 1μL/min and 5 μL/min.
