# Supplementary material for: Elasto-Inertial Focusing Mechanisms of Particles in Shear-Thinning Viscoelastic Fluid in Rectangular Microchannels
Source: Micromachines (Basel). 2022 Dec 1;13(12):2131. doi: 10.3390/mi13122131 (PMC9781382; doi:10.3390/mi13122131)
Supplement: Supplementary file 1 [file micromachines-13-02131-s001.zip › micromachines-2015279-supplementary.pdf]

Supplementary Information

Elasto-Inertial Focusing Mechanisms of Particles in Shear-Thinning Viscoelastic Fluid in Rectangular Microchannels

Mohammad Moein Naderi, Ludovica Barilla, Jian Zhou, Ian Papautsky*, Zhangli Peng*

University of Illinois Chicago, Department of Biomedical Engineering, Chicago, IL, 60607, USA

*Address correspondence to:

Dr. Ian Papautsky
Department of Biomedical Engineering
851 S. Morgan Street, 218 SEO
University of Illinois Chicago
Chicago, IL 60607, USA
E-mail: papauts@uic.edu; Tel: +1.312.413.3800

Dr. Zhangli Peng
Department of Biomedical Engineering
851 S. Morgan Street, 218 SEO
University of Illinois Chicago
Chicago, IL 60607, USA
E-mail: zhpeng@uic.edu; Tel: +1.312.996.8467

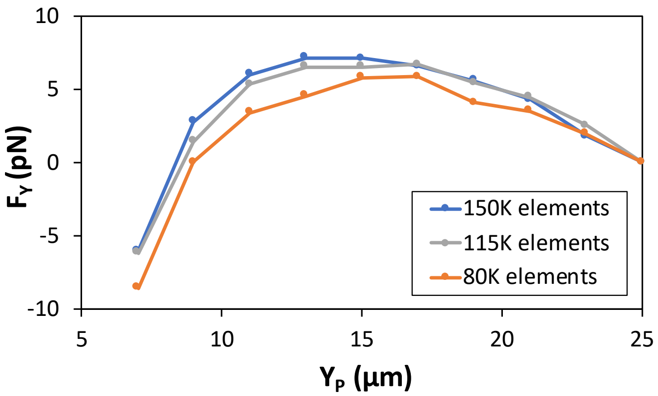

Fig S1. Mesh independence test results. Lateral force curves along the Y-midline at Q = 1µL/min for 80,000, 115,000, and 150,000 domain mesh elements.

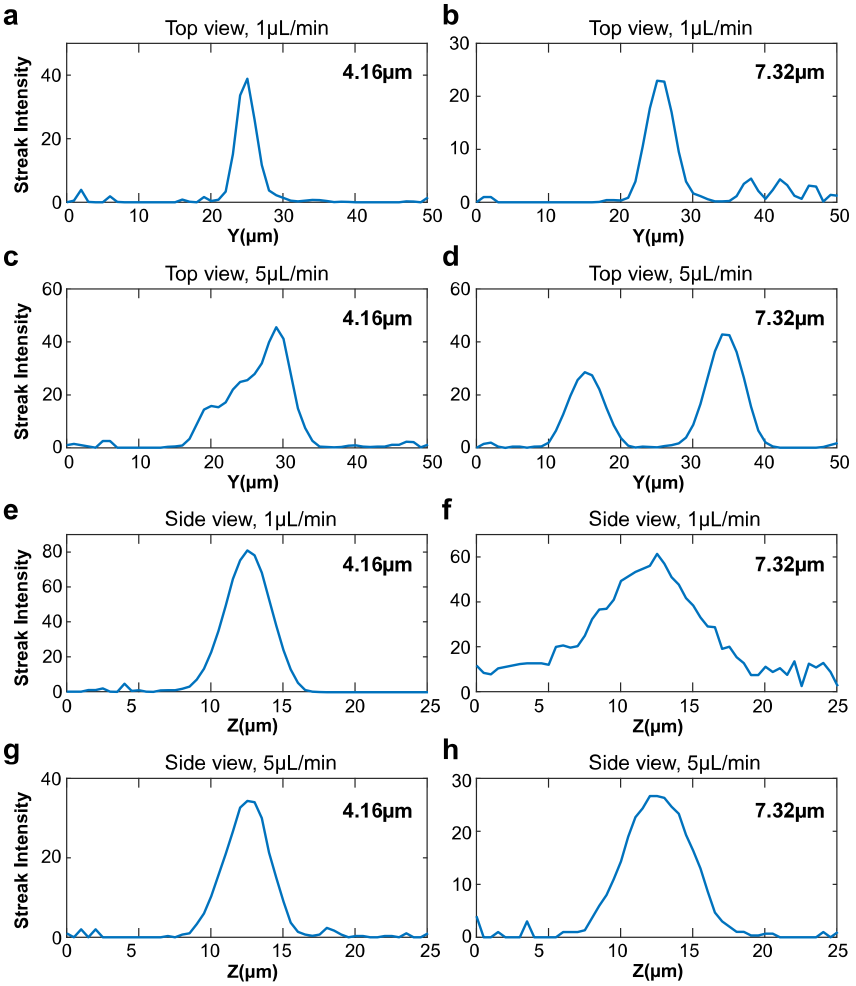

Fig S2. Line scan data of the fluorescent intensity of the focused streams for the 4.16µm and 7.32µm particles at (a,b) 1µL/min, top view, (c,d) 5µL/min, top view, (e,f) 1µL/min, side view, (g,h) 5µL/min, side view

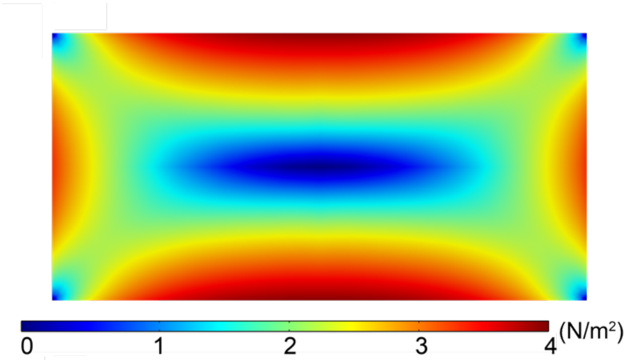

Fig S3. Simulation result of the first normal stress difference (N_1) at Q = 1μL/min. (*Re* = 0.2, *Wi* = 3.6, *El* = 18). Only a quarter of the channel is simulated due to symmetry and the results are mirrored about both axes of symmetry.

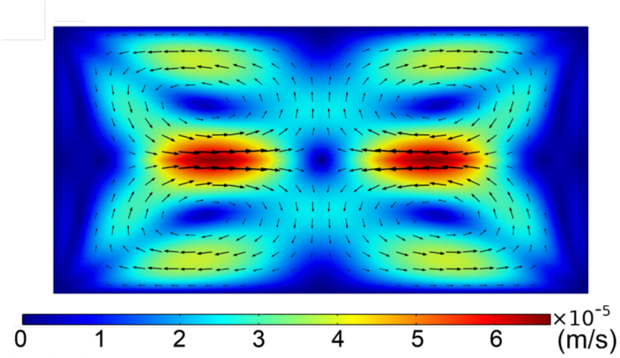

Fig S4. Simulation result of the magnitude and direction of the N_2-induced secondary flow at Q = 1μL/min. (*Re* = 0.2, *Wi* = 3.6, *El* = 18). Only a quarter of the channel is simulated due to symmetry and the results are mirrored about both axes of symmetry.

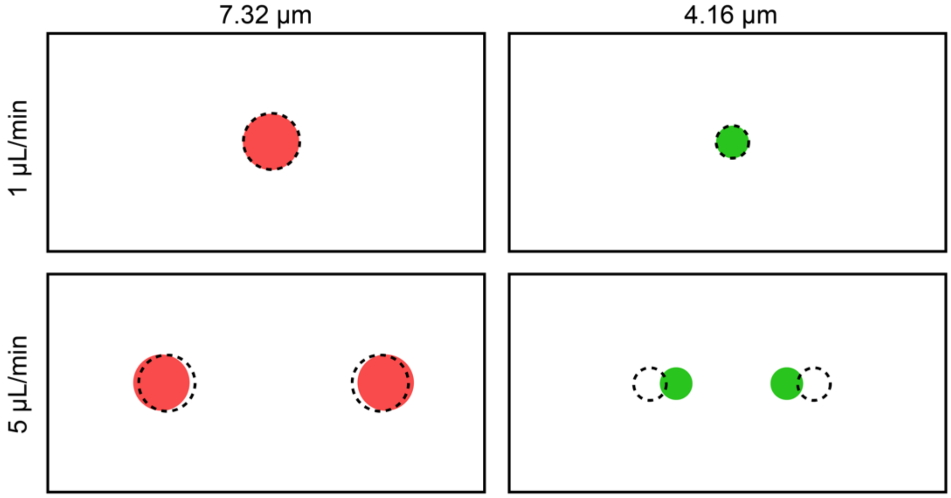

Fig S5. Channel cross-section schematic comparing the experimental (colored circles) observations and the simulation results (dashed circles) for the focusing position of 4.16μm and 7.32μm particles at 1μL/min and 5μL/min. At 1μL/min, simulation results predict the

focusing positions perfectly. At 5μL/min, the overall behavior of both particle sizes in the simulations is in agreement with the experiments. However, predicted focusing position for the 7.32μm particle is 1μm closer to the center, and the simulated focusing position for the 4.16μm particles is 4μm closer to the side walls.
